# An Intervention to Improve Medication Adherence in People With Heart Disease (Text4HeartII): Randomized Controlled Trial

**DOI:** 10.2196/24952

**Published:** 2021-06-09

**Authors:** Ralph Maddison, Yannan Jiang, Ralph Stewart, Tony Scott, Andrew Kerr, Robyn Whittaker, Jocelyn Benatar, Anna Rolleston, Paul Estabrooks, Leila Dale

**Affiliations:** 1 Institute for Physical Activity and Nutrition Deakin University Burwood Australia; 2 Department of Statistics The University of Auckland Auckland New Zealand; 3 Department of Cardiology Auckland District Health Board Auckland New Zealand; 4 Department of Cardiology Waitemata District Health Board Auckland New Zealand; 5 Section of Epidemiology and Biostatistics and School of Medicine University of Auckland Auckland New Zealand; 6 National Institute for Health Innovation University of Auckland Auckland New Zealand; 7 Waitemata District Health Board Auckland New Zealand; 8 The Centre for Health Tauranga New Zealand; 9 Department of Health Promotion, Social and Behavioral Health University of Nebraska Medical Centre Nebraska, NE United States

**Keywords:** cardiovascular disease, self-management, text messaging, risk factors

## Abstract

**Background:**

Mobile health technologies have the potential to improve the reach and delivery of interventions for promoting long-term secondary prevention of coronary heart disease.

**Objective:**

This study aims to determine the effectiveness of an SMS text messaging intervention (Text4HeartII) for improving adherence to medication and lifestyle changes over and above usual care in people with coronary heart disease at 24 and 52 weeks.

**Methods:**

A two-arm, parallel, randomized controlled trial was conducted in New Zealand. Participants with a recent acute coronary syndrome were randomized to receive usual cardiac services alone (control, n=153) or a 24-week SMS text message program for supporting self-management plus usual cardiac services (n=153). The primary outcome was adherence to medication at 24 weeks, defined as a medication possession ratio of 80% or more for aspirin, statin, and antihypertensive therapy. Secondary outcomes included medication possession ratio at 52 weeks, self-reported medication adherence, adherence to healthy lifestyle behaviors, and health-related quality of life at 24 and 52 weeks.

**Results:**

Participants were predominantly male (113/306, 80.3%) and European New Zealanders (210/306, 68.6%), with a mean age of 61 years (SD 11 years). Groups were comparable at baseline. National hospitalization and pharmacy dispensing records were available for all participants; 92% (282/306, 92.1%) of participants completed a 24-week questionnaire and 95.1% (291/306) of participants completed a 52-week questionnaire. Adherence with 3 medication classes were lower in the intervention group than in the control group (87/153, 56.8% vs 105/153, 68.6%, odds ratio 0.60, 95% CI 0.38-0.96; *P*=.03) and 52 weeks (104/153, 67.9% vs 83/153, 54.2%; odds ratio 0.56, 95% CI 0.35-0.89; *P*=.01). Self-reported medication adherence scores showed the same trend at 52 weeks (mean difference 0.3; 95% CI 0.01-0.59; *P*=.04). Moreover, self-reported adherence to health-related behaviors was similar between groups.

**Conclusions:**

Text4HeartII did not improve dispensed medication or adherence to a favorable lifestyle over and above usual care. This finding contrasts with previous studies and highlights that the benefits of text interventions may depend on the context in which they are used.

**Trial Registration:**

Australian New Zealand Clinical Trials Registry ACTRN12616000422426; http://www.anzctr.org.au/Trial/Registration/TrialReview.aspx?id=370398.

**International Registered Report Identifier (IRRID):**

RR2-10.1186/s13063-018-2468-z

## Introduction

### Background

Coronary heart disease (CHD) is a leading cause of premature death and disability worldwide [1]. Improved diagnosis, treatment, and management have substantially reduced the mortality rate of individuals living with CHD [2,3]. Following a cardiac event, clinical guidelines recommend people should participate in cardiac rehabilitation (CR), which is a multicomponent program that educates and supports self-management for the secondary prevention of CHD. CR aims to encourage people to make healthy lifestyle changes for reducing subsequent cardiac events. Lifestyle changes typically include initiating and maintaining regular physical activity, eating a healthy diet, stopping smoking, reducing harmful alcohol intake, and taking medications as per the prescribed regimen [4]. Appropriate self-management is critical for people with CHD to maximize treatment benefits [5].

Despite its benefits, participation in CR has been shown to be inadequate in all countries in which it has been measured [6]. In response, various alternative modes of delivery, including home-based CR and telehealth, have been developed, with similar reductions in cardiovascular risk factors as compared with hospital-based programs [7]. Most recently, mobile health technology has been used to better support the long-term self-management in people with cardiovascular disease [8]. Mobile health has the potential to be an extremely powerful tool for influencing behavior at a population level because it is widely available globally, inexpensive, and allows instant delivery of information [9].

SMS text messaging is the most widely used mobile phone intervention [10,11]. The strongest evidence for SMS text messaging in CHD is from the TEXT ME randomized controlled trial (RCT; n=710) [12], which reported statistically significant positive effects on low-density lipoprotein cholesterol, with more sizable effects on various secondary outcomes. Despite its effectiveness, the study was limited to a single center in Australia and excluded many participants because they did not own a mobile phone, which limited generalizability. TextMe was also evaluated as a standalone strategy; thus, it was unclear whether the intervention was more or less beneficial to those in traditional programs.

To address these limitations, we conducted the Text4HeartII trial across 2 district health boards in Auckland, New Zealand. This trial extended our previous Text4Heart randomized controlled pilot trial (n=123) [13,14], which found a doubling of adherence to lifestyle behaviors at 3 months but not at 6 months. Here, we present findings from a larger effectiveness trial of Text4Heart using an objective measure of medication adherence.

### Aims

This study aimed to determine the effectiveness of the Text4HeartII self-management program for improving adherence to medication and lifestyle changes in addition to usual care in people with an acute coronary syndrome (ACS) at 24 and 52 weeks.

## Methods

### Overview

A two-arm, parallel RCT was conducted in 2 large metropolitan hospitals in Auckland, New Zealand between July 2016 and November 2019. The study was approved by the New Zealand Health and Disability Ethics Committee (15/NTA/205), and the protocol was registered and published before the conclusion of recruitment (Australian New Zealand Clinical Trials Registry, ID: ACTRN12616000422426. Registered, April 1, 2016). The trial was developed and reported in accordance with the CONSORT (Consolidated Standards of Reporting Trials) statement (Multimedia Appendix 1). No changes were made to the methods after commencement of the trial.

### Participants

Eligible participants were adults with an ACS (including those who had undergone a percutaneous coronary revascularization procedure), clinically stable, able to read English, and able to provide informed consent. Participants were excluded if they had untreated ventricular tachycardia, severe heart failure, life-threatening coexisting disease with life expectancy of less than 1 year, and significant exercise limitations other than cardiovascular disease. Given the high level of mobile phone penetration (there were 6.5 million mobile connections in New Zealand in 2020; population 5.0 million people), mobile phone ownership was not considered in the eligibility criteria [15].

### Procedures

#### Overview

Research nurses recruited participants from 2 metropolitan hospitals in the Auckland region of New Zealand (Auckland City and North Shore Hospitals) before discharge from the hospital following an ACS or post discharge (within 6 weeks) via telephone. Potential participants were contacted in person to determine their interest in the study. Nurses undertook screening to determine their eligibility. Those who met the eligibility criteria were provided with a participant information sheet and consent form. For this trial, informed consent was obtained verbally or in person depending on when the participant agreed to participate. Interested participants contacted the research nurse to schedule a time for baseline assessments.

The trial was nested within the existing Australian and New Zealand Acute Coronary Syndrome-Quality Improvement (ANZACS-QI) program, which allowed routinely collected data to be accessed for baseline and follow-up assessments. ANZACS-QI [16] is a web-based app deployed nationally to securely gather data on every suspected patient with an ACS in New Zealand and is embedded in more than 90% of hospitals, including those involved in this study. ANZACS-QI provided in-hospital data on all people with an ACS, with risk stratification, diagnosis, investigation management, and complications [17]. Individual behavioral data (diet, exercise, alcohol consumption, and smoking status) were assessed via a telephone interview. In addition, during the 24-week follow-up call, participants were asked to respond to specific questions regarding their overall perceptions of the Text4HeartII program.

#### Sample Size

A total sample of 330 participants (165 per group) was estimated to provide 80% power at the 5% level of significance (two-sided) to detect an absolute between-group difference of 15% in the proportions of participants adherent to medication at the end of the 24-week intervention (assuming a control rate of 30%). The conservative control rate was based on self-reported medication data from our original Text4Heart study and on previous New Zealand research [18], which found that only 60% of patients had a medication possession ratio (MPR) >0.8 for statins; we believed that this value would be lower if all classes of medication (statins, antihypertensive, and antiplatelet therapy) were included.

#### Randomization, Allocation Concealment, and Blinding

Upon submission of baseline data, eligible participants were randomly allocated in a 1:1 ratio to the intervention or control groups using block randomization with variable block sizes of 2 or 4, stratified by hospital. The randomization sequence was generated by a biostatistician (YJ). Study investigators (but not participants) were blinded to the intervention allocation during the trial. Primary outcome data (prescribed medication) were obtained via the Ministry of Health National Data Linkage, thereby mitigating any bias associated with self-reported outcome assessments.

#### Control and Intervention

All participants received usual medical management and were offered center-based CR as per guidelines. CR offered at the participating hospital sites consisted of a 1-hour outpatient education program per week for 6 weeks at a hospital or community center covering a range of topics, such as cardiovascular risk factors, lifestyle changes, and psychosocial support. Patients were also encouraged to attend a 16-session supervised exercise program at the participating hospital or outpatient center. Participants could also participate in usual care CR from the point of discharge to 24 weeks after their cardiac event. In addition to usual care, the intervention group received a 24-week program of automated daily SMS text messages commencing within a week of the baseline assessment. All participants were telephoned at 24 and 52 weeks post randomization to obtain follow-up self-reported outcome data.

#### Intervention

Text4HeartII comprised a personalized, automated program of self-management that was delivered via SMS text messages over 24 weeks (full details are provided in the study protocol) [19]. The overall goal of the intervention was to have individuals adhere to the New Zealand treatment guidelines for an ACS [20]. Specifically, Text4HeartII included core *Heart Health* content comprising education and support to encourage regular taking of medication, eat a healthy diet (including moderating alcohol consumption), manage stress, and exercise regularly (total 126 messages). Additional SMS text messages were delivered based on the suboptimal behavior participants wanted to modify (eg, physical activity, heart healthy diet, stress management, and stop smoking); each module contained 35 text messages. Modules were discussed with research nurses at baseline to identify participants’ preferences. Participants were only able to choose one additional module; however, smokers were prioritized to receive messages providing cessation support. All content was grounded in established psychological (Common Sense Model) [21] and behavior change (social cognitive) theory [22]. The content was focused on modifying people’s perceptions of the symptoms, timeline, cause, consequences, personal control over, and the ability of treatment to prevent cardiovascular disease [23] as well as altering the key mediators of behavior change, including self-efficacy, social support, and motivation. The intervention content was based on the original Text4Heart pilot program, with some modifications. In Text4HeartII, we did not include a user website, as this feature was seldom used in the original pilot study [14]. We also revised the message content from weeks 12 to 24 to promote maintenance of the behaviors and relapse prevention.

Participants received a minimum of 1 core heart message per day for 24 weeks, with an additional 35 messages sent over the first 12 weeks; all messages were sent from a centralized server. Messages were sent at times to suit the participants and were personalized with the participant’s name. Messages were predominantly unidirectional, but participants were able to text the research team to share their progress if they wanted (eg, goals achieved). Brief training was offered to all participants at enrollment on how to read a text message and how to delete or save messages. No changes were made to the intervention content or delivery during the study period. Examples of the SMS text messages are provided in Textbox 1.

**Examples of SMS text messages.**

**Heart Health**

- T4H: Know your numbers – when is the last time you’ve had your cholesterol or blood pressure checked? Ask next time you see your GP.
- T4H: High cholesterol or high blood pressure is not good for your heart condition. Your medications will help improve these.
- T4H: Think about your future health. How do you want to feel in 6 months? Try setting small goals with your GP or support person to reach that.

**Medication**

- T4H: It can be scary to think about the chance of having another heart problem. Taking your pills and changing your lifestyle can lower the risk.
- T4H: It’s important to take your medications regularly. To help remember make this part of your daily routine, such as after brushing your teeth.

**Physical Activity**

- T4H: Changing it up can improve your fitness. Do long slow walks, then head for some hills, then hit the track for speed. Start slow & build.
- T4H: Sometimes, it is hard to exercise when it is raining, try an indoor activity or grab an umbrella and wrap up.

**Diet**

- T4H: Be wary of low-fat food. Not all are good for you, some are still high in sugar. Read your food labels and compare using the 100g column.
- T4H: To increase your servings of fruit & veg, add a can of tomatoes to your dish or pour frozen veggies into stews/soups/risottos.

**Smoking**

- T4H: Draw a habit map. When I smoke at work its because..., when I smoke at home its because..., think of the reasons, fix the causes!
- T4H: Find yourself a quit buddy who you can call or txt if you get down. You can make each other feel better with encouragement & share tips.

**Stress and Relaxation**

- T4H: If you feel discomfort after your heart event, such as feeling angry, sad or withdrawn, consider talking to a health professional.
- T4H: If you feel stressed, close your eyes and imagine a scene where you feel calm. It might be a tropical beach, a forest, or a favorite spot.

#### Outcomes

All outcomes were assessed at 24 and 52 weeks post randomization. The primary outcome was patient adherence to prescribed medication at 24 weeks, defined as an MPR of 80% or more for 3 medication classes, namely, antiplatelet agent (aspirin), statin, and antihypertensive therapy (angiotensin-converting enzyme inhibitor [ACEI] or angiotensin receptor blocker [ARB] and/or a β-blocker), consistent with the guideline-recommended therapy [20]. To obtain the primary outcome, MPR was calculated for each drug class and then combined to determine adherence across the 3 combined classes. The choice of primary outcome was driven by the positive effect observed on self-reported medication adherence at 6 months in our pilot study and by the ability to provide an objective assessment of medication adherence (namely MPR). The MPR approach has been used successfully in New Zealand to assess statin use [18]; we adapted this approach for the other medication classes. MPR reflects the number of days the drug was assumed to be in a patient’s possession (based on dispensed drugs) divided by the number of days spent out of hospital and alive over the assessment period. In a previous study in New Zealand, the possession of medications during 80% or more of follow-up time (ie, MPR ≥0.8) was indicative of maintenance [18]. This proportion was based on post hoc analyses from previous trials on the effect of statin compliance on coronary events and all-cause mortality [24]. To calculate the MPR, community pharmacy dispensing records were linked using the encrypted National Health Index (NHI) via the National Pharmaceuticals Collection database.

### Secondary Outcomes

#### Objective Secondary Outcomes

The MPR for each class of medication (aspirin, statins, ACEI/ARB, and/or β-blockers) was also assessed at 52 weeks using the same approach as used at 24 weeks. Blood pressure, total cholesterol, low-density lipoprotein cholesterol, and high-density lipoprotein cholesterol data were obtained from the ANZACS-QI registry; these were based on routinely collected data.

Self-reported outcomes were measured by a trained research assistant during a telephone call at 24 and 52 weeks. Self-reported medication adherence was assessed using the Morisky 8-item Medication Adherence Scale [25] (0=high, 1-2=medium, and 3-6=low adherence). A license for use was obtained in this study. Adherence to recommended lifestyle behaviors was measured using a composite health behavior score adapted from the European Prospective into Cancer–Norfolk prospective population study [26]. For this study, we used New Zealand–relevant questions to capture all 4 health behaviors. This approach differed slightly from the European Prospective into Cancer–Norfolk study, which used plasma vitamin C >50 mmol/L to indicate fruit and vegetable intake of at least five servings per day. In our study, the following measures were used to determine participants’ health behavior scores:

1. Smoking status was measured using 3 items from a validated smoking history questionnaire [27] and included “have you ever smoked, have you had a puff in the last week and when they quit smoking” (if appropriate).
2. Physical activity level was assessed using items adapted from the New Zealand Health Survey to assess the daily time spent in light-, moderate-, and vigorous-intensity activities [28]. The time spent in moderate-to-vigorous physical activity (MVPA) was calculated and used to indicate adherence to guidelines [29].
3. Alcohol consumption was measured using the alcohol use disorders identification test of alcohol consumption questions [30], a screening tool designed to assess the units of alcohol consumed per week and to identify people who are hazardous drinkers. Index cards referencing standard drink sizes were used to reduce comprehension errors.
4. Fruit and vegetable intake were assessed by 2 New Zealand–specific questions used in the 2006/2007 New Zealand Health Survey (N=12,488, including adults with CHD) [28].

Participants received a score on a 4-point scale for each of the key risk factors, with 1 point each assigned for being a current nonsmoker, meeting physical activity guidelines to achieve some health benefits (≥150 min of MVPA per week), consuming 14 or fewer standard alcoholic drinks per week, and consuming at least five servings of fruits and vegetables per typical day. A total score of 3 or 4 was considered adherence to healthy behaviors. No changes were made to study the outcomes once the trial commenced.

Health-related quality of life was assessed using the European Quality of Life 5 dimensions [31] measure of health status.

#### Perceptions of Text4HeartII

During the 24-week follow-up telephone call, participants were asked to respond to specific questions about Text4HeartII. Questions were based on those used in the Text4Heart pilot trial and included their perceptions of the program, technical issues experienced, and whether they changed behaviors. Participants who responded that they had changed behaviors were asked to indicate which specific behaviors they had changed.

#### Adverse Events

All participants were telephoned at 24 and 52 weeks by a trained researcher to determine whether the participants had experienced any serious adverse events during the course of the study. Serious adverse events were reported to a registered medical physician to determine whether they were associated with the study treatment and to determine the course of action (if needed).

#### Statistical Analysis

Trial data collected from all eligible participants were linked to the national database using encrypted NHIs for the purpose of analysis. Treatment evaluations were performed according to the intention-to-treat principle. There were no missing data on the MPR (primary outcome), which were obtained via national data linkage. Continuous variables were summarized as mean and SD, and categorical variables were summarized as frequency and percentage. Logistic regression was conducted to evaluate the main treatment effects on medication adherence at 24 and 52 weeks, adjusting for hospital (stratification factor). Odds ratios (ORs) and associated 95% CI were reported at each visit. The same regression models were used for adherence to healthy behaviors at 24 and 52 weeks. The analysis of covariance regression model was used to evaluate the treatment effect on continuous secondary outcomes, adjusting for the baseline value and hospital. Model-adjusted mean differences were reported with 95% CI. Statistical analysis was performed using SAS version 9.4 (SAS Institute Inc.). All statistical tests were two-sided at a 5% significance level.

## Results

### Overview

**Figure 1:**
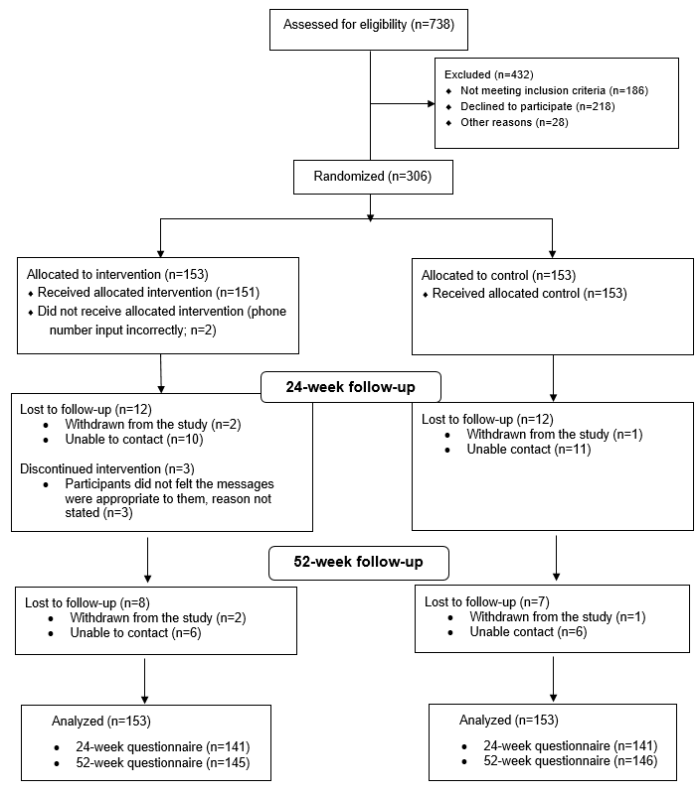
Participant flow.

Figure 1 presents the flow diagram of the progress through the phases of the trial. A total of 739 people were screened between July 2016 and September 2018. The last trial participant completed the 24-week follow-up on October 2, 2019. Study data collection was completed in November 2019. A total of 306 eligible participants were randomized (153 per group), 282 participants were followed up at 24 weeks (141 per group), and 291 participants were followed up at 52 weeks (intervention: n=145 and control: n=146). All participants had MPR data at both 24 and 52 weeks, which were obtained directly from national medication dispensing records using the linked encrypted NHIs.

The participants were predominantly male (113/306, 80.3%) and New Zealand European (210/306, 68.6%), with a mean age of 61 years (SD 11) years. The groups were comparable at baseline (Table 1). Most participants were employed full-time (115/306, 37.6%) or retired (132/306, 43.1%). Most participants were married or living with a partner (230/306, 75.2%).

**Table 1:** Baseline demographic, clinical, and behavioral data of all randomized participants (N=306).

Characteristic					Control (n=153)			Intervention (n=153)
**Demographics**								
	Age (years), mean (SD)			61 (11)			61 (11)	
	Male, n (%)			113 (73.8)			123 (80.4)	
	**Ethnicity, n (%)**							
		New Zealand European	102 (66.7)			108 (70.6)		
		Maori	11 (7.2)			12 (7.8)		
		Pacific	8 (5.2)			5 (3.3)		
		Asian	13 (8.5)			11 (7.2)		
		Other	19 (12.4)			17 (11.1)		
	**Employment, n (%)**							
		Self-employed	33 (21.6)			29 (18.9)		
		Full-time employment	55 (35.9)			60 (39.2)		
		Part-time employment	9 (5.9)			5 (3.3)		
		Retired	46 (30.1)			46 (30.1)		
		Other (full-time homemaker, student, unemployed, or beneficiary)	10 (6.5)			13 (8.5)		
	**Marital status, n (%)**							
		Married or living with partner	124 (81)			106 (69.3)		
		Separated, divorced, or widowed	24 (15.7)			33 (21.6)		
		Never married	5 (3.3)			14 (9.1)		
**Clinical data, mean (SD)**								
	BMI (kg/m^2^)			29.21 (5.6)			29.74 (5.9)	
	**Cholesterol (mmol/L)**							
		Total	4.74 (1.2)			5.07 (1.3)		
		HDL-C^a^	1.19 (0.3)			1.20 (0.5)		
		LDL-C^b^	2.63 (1.1)			2.99 (1.2)		
	Systolic blood pressure (mm Hg)			129 (16)			129 (18)	
	Diastolic blood pressure (mm Hg)			74 (10)			75 (13)	
**Behavioral risk factors**								
	Meeting MVPA^c^ guidelines, n (%)			70 (46)			75 (49)	
	Not smoking, n (%)			140 (91)			138 (90)	
	Number of drinks ≤14 per week, n (%)			132 (86)			133 (87)	
	Fruit and vegetables serves ≥5 week, n (%)			84 (55)			71 (46)	
	HRQoL^d^—health state score, mean (SD)			64 (22)			62 (22)	

^a^HDL-C: high-density lipoprotein cholesterol.

^b^LDL-C: low-density lipoprotein cholesterol.

^c^MVPA: moderate-to-vigorous physical activity.

^d^HRQoL: health-related quality of life.

Adherence to the 3 medication classes (aspirin, statin, and any antihypertensive) in the intervention group was worse than that in the control group at 24 weeks (OR 0.60, 95% CI 0.38-0.96; *P*=.03). Differences in adherence to all 4 medication classes (aspirin, statin, β-blocker, and ACEI/ARB; OR 0.67, 95% CI 0.42-1.05; *P*=.08) and for individual classes of aspirin, statins, and β-blockers (Table 2) were not statistically significant, except for adherence to ACEI/ARB (OR 0.49, 95% CI 0.30-0.82; *P*=.006), which was worse in the intervention group than in the control group.

**Table 2:** The effect of intervention on medication adherence at 24- and 52-week follow-up (full cohort).

Adherence to	24 weeks (N=306)					52 weeks (N=306)			
	Control (n=153), n (%)	Intervention (n=153), n (%)	Adjusted OR^a^ (95% CI)	*P* value	Control (n=153), n (%)		Intervention (n=153), n (%)	Adjusted OR (95% CI)	*P* value
All 3 drug classes (aspirin, statin, and any BP^b^-lowering drug): primary outcome	105 (68.6)	87 (56.8)	0.60 (0.38-0.96)	.03	104 (67.9)		83 (54.2)	0.56 (0.35-0.89)	.01
All 4 drug classes (aspirin, statin, β-blocker, and ACEI^c^/ARB^d^)	71 (46.4)	56 (36.6)	0.67 (0.42-1.05)	.08	70 (45.7)		56 (36.6)	0.68 (0.43-1.08)	.11
Statin	131 (85.6)	122 (79.7)	0.66 (0.36-1.20)	.18	129 (84.3)		119 (77.7)	0.65 (0.36-1.16)	.15
Aspirin	124 (81.0)	122 (79.7)	0.92 (0.52-1.62)	.78	123 (80.3)		119 (77.7)	0.85 (0.49-1.49)	.58
β-Blocker	103 (67.3)	100 (65.3)	0.92 (0-1.48)	.73	102 (66.6)		89 (58.1)	0.69 (0.43-1.11)	.13
ACEI/ARB	119 (77.7)	97 (63.4)	0.49 (0.30-0.82)	.006	123 (80.3)		97 (63.4)	0.42 (0.25-0.71)	.001
BP-lowering drugs (ACEI/ARB and/or β-blocker)	137 (89.5)	122 (79.7)	0.46 (0.24-0.88)	.02	139 (90.8)		113 (73.8)	0.28 (0.15-0.55)	<.001

^a^OR: odds ratio; odds ratio compares the estimated odds between intervention and control groups.

^b^BP: blood pressure.

^c^ACEI: angiotensin-converting enzyme inhibitor.

^d^ARB: angiotensin II receptor blockers.

Similar results were found for medication adherence at 52 weeks (secondary outcome), with higher adherence to 3 medication classes in the control group than in the intervention group (OR 0.56, 95% CI 0.35-0.89; *P*=.01). The difference in adherence to the 4 medication classes was not significant (OR 0.68, 95% CI 0.43-1.08; *P*=.10). Similarly, there were no significant differences for individual classes of aspirin, statins, and β-blockers (Table 2), but adherence to ACEI/ARB was less in the intervention group than in the control group (OR 0.42, 95% CI 0.25-0.71; *P*=.001). These data are mirrored to some extent by the self-reported medication adherence data. At 24 weeks, there was a small effect on self-reported medication adherence, which favored the control group (adjusted mean difference 0.15; 95% CI −0.15 to 0.45; *P*=.33), with a larger effect observed at 52 weeks (adjusted mean difference 0.30; 95% CI 0.01-0.59; *P*=.04.

The composite measure of adherence with lifestyle behaviors was similar for both the intervention and control groups at 24 weeks (OR 1.11, 95% CI 0.65-1.90; *P*=.70) and 52 weeks (OR 0.97, 95% CI 0.58-1.62; *P*=.90; Table 3). In terms of individual behaviors, the number of participants who did not smoke at 24 weeks was higher in the intervention group (n=138) than in the control group (n=132; OR 5.75, 95% CI 1.08-30.61; *P*=.04). No other differences were observed between these groups. At 52 weeks, there was a trend for differences in time spent in vigorous-intensity activity (mean difference 44.8 min; 95% CI −3.9 to 93.5; *P*=.07) and MVPA (mean difference 100.3 min; 95% CI −16.6 to 217.3; *P*=.09), which favored the intervention group. A total of 75 serious adverse events were reported during the trial; however, none of them were related to the study. Only 1 participant died during the study period.

**Table 3:** The effect of intervention on adherence to lifestyle risk factors (secondary outcomes) at 24- and 52-week follow-up.

Adherence to	24 weeks (n=282)							52 weeks (n=291)					
	Control		Intervention		Adjusted OR^a^ (95% CI)	*P* value	Control			Intervention		Adjusted OR (95% CI)	*P* value
	Sample size, n	Participant, n (%)	Sample size, n	Participant, n (%)			Sample size, n		Participant, n (%)	Sample size, n	Participant, n (%)		
Lifestyle behaviors (composite)	141	94 (66.7)	141	100 (70.9)	1.11 (0.65-1.90)	.70	146		95 (65.1)	145	96 (66.2)	0.97 (0.58-1.62)	.90
Physical activity guideline	141	68 (48.3)	140	79 (56.4)	1.43 (0.87-2.35)	.16	146		80 (54.8)	145	88 (60.7)	1.25 (0.75-2.09)	.39
Smoking cessation	141	132 (93.6)	141	138 (97.8)	5.75 (1.08 -30.61)	.04	146		139 (95.2)	145	139 (95.7)	1.38 (0.39-4.88)	.62
Low alcohol consumption	140	132 (94.3)	141	130 (92.2)	0.68 (0.24-1.87)	.45	145		141 (97.2)	145	136 (93.8)	0.33 (0.09-1.28)	.11
Fruit and vegetable guidelines	141	67 (47.5)	141	64 (45.4)	1.01 (0.61-1.66)	.98	144		64 (44.4)	145	54 (37.2)	0.81 (0.49-1.33)	.40

^a^OR: odds ratio; odds ratio compares the estimated odds between intervention and control groups.

### Perceptions of Text4HeartII

Table 4 shows participants’ responses to questions regarding the Text4HeartII intervention. Overall, most participants (136/139, 97.8%) did not experience any technical issues and would recommend the program to others. Most (114/138, 82.6%) participants felt that the program helped them manage their heart disease, but perceptions of whether the program influenced the behavior were mixed.

**Table 4:** Participants’ responses on Text4HeartII (n=139).

Question					Sample size, n			Participants, n (%)
“Did you have any technical problems with the program?”					139			136 (97.8)
“Would you recommend the program to other people who have had a heart event?”					138			134 (97.1)
“Did taking part in this program help you learn about heart condition?”					138			86 (62.3)
“Did taking part in this program help you in the recovery from your heart condition?”					138			114 (82.6)
“**Did taking part in this program help you change your behaviors?”**					138			78 (56.5)
	“**If yes, which behaviors did you change?”**			78				
		“I became physically active”				41 (52.5)		
		“I ate more fruit and vegetables”				34 (43.6)		
		“I ate less saturated fat”				21 (26.9)		
		“I took my medication regularly”				19 (24.3)		
		“I drank less alcohol”				10 (12.8)		
		“I ate less salt”				10 (12.8)		
		“I lowered my level of stress”				9 (11.5)		
		“I lost weight”				7 (9.0)		
		“I stopped smoking”				5 (6.4)		
		“I ate more healthy fat”				4 (5.1)		
		“I had regular GP^a^ checks”				3 (3.8)		
		“I watched less TV^b^”				0 (0)		
		“I got more adequate sleep”				0 (0)		

^a^GP: general practitioner.

^b^TV: television.

## Discussion

### Principal Findings

The Text4HeartII trial extended our pilot trial to determine the effectiveness of an SMS text messaging–based intervention to improve adherence to medication and lifestyle behaviors at both 24 and 52 weeks. Overall, we found no evidence to support the effectiveness of the program on dispensed medication or on adherence to a composite measure of lifestyle change over and above usual care.

The strengths of this trial were the RCT design and the objective assessment of medication adherence. Our study addressed criticisms of previous SMS text messaging trials [32,33], including enhanced generalizability. Our trial was of sufficient duration to elicit behavior change, and the follow-up assessments (24 and 52 weeks) were long enough to determine the sustained effect of the intervention. An additional strength of this trial was the complete data on the primary outcome. A possible limitation could be using MPR as an outcome, which reflects the number of days a drug was assumed to be in a person’s possession (based on dispensed drugs) rather than a measure of whether a person took their medication or not. Nevertheless, previous studies have used MPR as a proxy measure of medication adherence [20].

### Comparison With Other Studies

The lack of a positive effect on mediation adherence was surprising and contrary to previous research. A meta-analysis of 16 RCTs (N=2742) showed that SMS text messaging significantly improved medication adherence across a range of conditions (OR 2.11, 95% CI 1.52-2.93; *P*<.001) [34]. The effect was not sensitive to study characteristics (intervention duration or type of disease) or text message characteristics (personalization, 2-way communication, or daily text message frequency). The most commonly used method to assess adherence was self-report (9 studies), followed by the medication event monitoring system (4 studies).

Our findings differ from previous SMS text messaging trials in people with cardiovascular disease. A recent review (Text2PreventCVD) involving 9 trials (N=3779) and individual participant data (5 trials; n=2612) meta-analysis of SMS text messaging interventions demonstrated positive effects across a host of risk factors (BMI, systolic blood pressure, and diastolic blood pressure) [35]. Without directly comparing the messages within the respective interventions, it is difficult to know where the differences in effect may exist. However, a recent systematic description and comparison of the development processes of the interventions included in that systematic review highlighted that the Text4HeartII development process was comparable with that of previous trials [36]. Moreover, our Text4Heart pilot trial was included in the Text2PreventCVD systematic review and comparison of development processes. Like similar trials, the development of Text4HeartII involved consultation with experts, users, or other stakeholders; was based on literature reviews; included relevant theory; and involved primary research with end users [36]. The behavior change techniques used in this study were similar to those used in previous studies [36].

Given the existing body of evidence supporting SMS text messaging on medication adherence, it is clear that Text4HeartII did not have the desired effect on the population and setting in this trial. This may be attributed to personal factors associated with our cohort; randomized participants appeared to be more motivated to adhere than those in previous studies. The results showed that the difference between the control and intervention groups was relatively small and driven predominantly by MPR for antihypertensive medication. Despite similar days in hospital between groups at 24 weeks (approximately 1 day), adherence to all medication classes for both groups was higher than our proposed control rate (30%); approximately 41.5% (127/306) of the participants had an MPR >0.8. Our original control rate was based on previous research, which found that 59.3% (8028/13,520) of patients had an MPR >0.8 for statins only [18]. However, in this study, adherence to statins was higher; 82.6% (253/306) of participants had an MPR >0.8. Second, in our study, participants were recruited by cardiac nurses, and all participants were offered CR services, with good access to follow-up. The lack of marked differences between groups may have reflected organizational factors with higher levels of available support (including CR) at the 2 participating metropolitan hospitals. Thus, the effectiveness of SMS text messaging interventions may be strongly influenced by the population and context in which they are applied. This highlights the need to better understand the context in which SMS text messaging interventions are delivered and how individual- and organizational-level factors may affect the adoption, implementation, and effectiveness of these types of interventions. It also emphasizes the importance of evaluating interventions in the setting they are likely to be used before widespread adoption. Third, it is also possible that our text messages were not potent enough to evoke changes in medication adherence over and above usual cardiac care. Many previous SMS text messaging interventions have targeted single behaviors, such as smoking behavior, exercise, and medication adherence [32,34,37,38]. Thus, the lack of potency may have resulted from our approach to target multiple behaviors rather than targeting medication adherence alone. This is consistent with a previous qualitative research, which showed that conversations about changing multiple health behaviors were perceived to be overwhelming for patients and difficult to implement for health care professionals [39]. The lack of potency hypothesis is in line with qualitative responses from participants in this study, which showed that although 82.6% (114/138) of intervention participants responded that participating in this program had helped them recover from their heart condition, only 24.3% (19/78) felt the messages helped them to take their medications regularly. Furthermore, 56.5% (78/138) of the intervention participants responded that the program helped them change their health-related behaviors. Fourth, it is possible that the SMS text messaging program did not fully address the needs of participants [40]. Previous authors have highlighted the need for more personalized messages, depending on diagnosis (atherosclerosis, spontaneous coronary artery dissection, and Takotsubo cardiomyopathy) and key risk factors such as current smoking status. Although the proposed message library developed by Marshall et al [40] was similar to ours in terms of advice on a graduated exercise program, nutrition, smoking cessation, stress management, and medication, their proposed content also included messages around support groups and information about respective diagnoses. Patients could also send a message, which prompted a response from a CR nurse. The results of that study have not been published and were therefore not available for direct comparison with this study.

Surprisingly, the effects observed on lifestyle behaviors differed from those in our original pilot study [14]. In this study, we showed no clear differences in our composite measure of lifestyle change or individual behaviors, beyond those reported. The lack of effect may be attributed to the following issues. First, it is possible that there was a ceiling effect, with approximately 59.1% (181/306) of participants reporting adherence to 3 or more lifestyle behaviors at baseline. This could reflect self-reported bias, which was evident in the measurement of physical activity. Second, our measure of physical activity in this study differed from that in the pilot study. In this study, we used 3 items to capture the time spent in light-, moderate-, and vigorous-intensity activities. This approach was used to provide a better metric for adherence to guidelines for physical activity in adults (≥150 min of MVPA per day). Descriptive data suggested substantial overreporting of physical activity levels at all time points. For example, participants at baseline in both groups reported more than 300 minutes of daily MVPA, which is considerably higher than the standard population estimate for adults in New Zealand. Third, the lack of effect may have been related to the fact that both groups had well-managed risk factors at baseline (eg, few smokers, well-managed blood pressure), which limited the potential for change. Fourth, the lack of effect may also be attributed to changes made from the original intervention, which involved removing the website, which allowed people to set goals, review previous text messages, and access other resources related to CHD. We decided to remove the website component as it was infrequently used, and fewer than half of the participants in the pilot study felt using a website was a good way to deliver the program [14]. In the original study, we also issued pedometers and allowed people to track step counts, which was not a feature of this study. These features may have allowed for more interaction with the program but were removed because of pragmatic reasons (costs and distribution) related to the potential to scale as a national program.

### Future Research

Despite the lack of effect observed in our trial, SMS text messaging as an intervention has the potential to improve outcomes in people with CHD and other conditions. Findings from this study suggest that the context in which SMS text messaging interventions are delivered is important to consider and may have a significant impact on whether an intervention is effective or not. Future studies need to explore both individual- and organizational-level factors that may affect the adoption, implementation, and effectiveness of such interventions. For example, the RE-AIM (Reach, Effectiveness, Adoption, Implementation and Maintenance) framework [41,42] is a widely used evaluative framework for guiding the evaluation and reporting of health intervention effectiveness. It emphasizes collecting information about the reach, effectiveness, adoption, implementation, and maintenance of an intervention across both individual- and setting- or staff-level variables. RE-AIM aligns with systems-based approaches and allows for the assessment of vertical (eg, adoption decisions within a given organization) and horizontal (eg, adoption across different sectors) components. The application of RE-AIM or a similar framework would help in providing a clear understanding of the key factors that may affect the effectiveness of future text-based messaging interventions.

### Conclusions

There was no evidence to support the effectiveness of Text4HeartII on dispensed medication or adherence to a favorable lifestyle over and above usual care. In its current form, Text4HeartII cannot be used to augment existing services. The findings of this study are in contrast with those of previous studies and highlight the importance of evaluating interventions in the setting they are likely to be used before widespread adoption. Changes to the intervention program are warranted to justify its future implementation.

## Appendix

Multimedia Appendix 1 CONSORT (Consolidated Standards of Reporting Trials) statement checklist.

## Abbreviations

ACEI: angiotensin-converting enzyme inhibitor
ACS: acute coronary syndrome
ANZACS-QI: Australian and New Zealand Acute Coronary Syndrome-Quality Improvement
ARB: angiotensin receptor blocker
CHD: coronary heart disease
CONSORT: Consolidated Standards of Reporting Trials
CR: cardiac rehabilitation
MPR: medication possession ratio
MVPA: moderate-to-vigorous physical activity
NHI: National Health Index
OR: odds ratio
RCT: randomized controlled trial
RE-AIM: Reach, Effectiveness, Adoption, Implementation and Maintenance
